# Supplementary material for: EnzML: multi-label prediction of enzyme classes using InterPro signatures
Source: BMC Bioinformatics. 2012 Apr 25;13:61. doi: 10.1186/1471-2105-13-61 (PMC3483700; doi:10.1186/1471-2105-13-61)
Supplement: Addtional file 5 — The Java code to format the data files, evaluate and predict. The file enzml_java_code.tar.gz contains the Java code used to format database data to ARFF and XML formats, to execute cross and train-test (jackknife) evaluations and to record evaluation results to database. More information is included in the readme.txt file and the Javadoc files. The code can be used with a MySQL database. To use a different database software, other JDBC drivers might be required. [file 1471-2105-13-61-S5.gz › java_code/utils/doc/edu/cornell/lassp/houle/RngPack/Ranlux.html]

Ranlux


---


|  |  |  |
| --- | --- | --- |
| **Overview** \| **Package** \| **Class** \| **Use** \| **Tree** \| **Deprecated** \| **Index** \| **Help** | |  |
| **PREV CLASS**   **NEXT CLASS** | **FRAMES**    **NO FRAMES**     **All Classes** |  |
| SUMMARY: NESTED \| FIELD \| CONSTR \| METHOD | DETAIL: FIELD \| CONSTR \| METHOD |  |


---


## edu.cornell.lassp.houle.RngPack Class Ranlux

```
java.lang.Object
  cern.colt.PersistentObject
      edu.cornell.lassp.houle.RngPack.RandomElement
          edu.cornell.lassp.houle.RngPack.RandomSeedable
              edu.cornell.lassp.houle.RngPack.Ranlux
```

**All Implemented Interfaces:**: java.io.Serializable, java.lang.Cloneable

---

``` public class Ranlux extends RandomSeedable ```

RANLUX is an advanced pseudo-random number generator based on the
RCARRY algorithm proposed in 1991 by Marsaglia and Zaman.
RCARRY used a subtract-and-borrow algorithm with a period on the
order of 10^171^ but still had detectable correlations between
numbers. Martin Luescher proposed the RANLUX algorithm in 1993;
RANLUX generates pseudo-random numbers using RCARRY but
throws away numbers to destroy correlations. Thus RANLUX trades execution
speed for quality; by choosing a larger luxury setting one gets better random
numbers slower. By the tests availible at the time it was proposed,
RANLUX at the default luxury setting appears to be a significant
advance quality over previous generators.
  
  


|  |  |  |
| --- | --- | --- |
| LUXURY LEVELS | | |
| level | p |  |
| 0 | 24 | equivalent to the original RCARRY of Marsaglia and Zaman, very long period, but fails many tests. |
| 1 | 48 | considerable improvement in quality over level 0, now passes the gap test, but still fails spectral test. |
| 2 | 97 | passes all known tests, but theoretically still defective. |
| 3 | 223 | DEFAULT VALUE. Any theoretically possible correlations have very small chance of being observed. |
| 4 389 | highest possible luxury, all 24 bits chaotic. | |  |  |

   
 **VALIDATION** 
The Java version of RANLUX has been verified against published
values of numbers 1-5 and 101-105 produced by the reference implementation of
RANLUX for the following initial conditions:

- Default initialization: `Ranlux()`- Initialization with: `Ranlux(0,0)`- Initialization with: `Ranlux(389,1)`- Initialization with: `Ranlux(75,0)`

References:

- M. Luscher,  Computer Physics Communications **79** (1994)
  100- F. James, Computer Physics Communications **79** (1994) 111- About RANLUX random number generator: Excerpts from discussion in
      the Usenet news groups- Miller's FORTRAN 90 implementation of RANLUX with test code

Source code  is available.

**Version:**
:   1.0

**Author:**
:   Paul Houle  (E-mail: houle@msc.cornell.edu)

**See Also:**: Serialized Form

---

| **Field Summary** | |
| --- | --- |
| `static int` | `lxdflt`             Default luxury level: `lxdflt=3` |
| `static int` | `maxlev`             Maximum luxury level: `maxlev=4` |


**Constructor Summary**

`Ranlux()`
  
          Default initialization of RANLUX.

`Ranlux(java.util.Date d)`
  
          Initialize RANLUX with default luxury
level and a Date object.

`Ranlux(int ins)`
  
          Initialize RANLUX with default luxury
level and a specified seed.

`Ranlux(int lux,
java.util.Date d)`
  
          Initialize RANLUX with specified luxury
level and a Date object.

`Ranlux(int lux,
int ins)`
  
          Initialize RANLUX with specified luxury
level and seed.

`Ranlux(int lux,
long ins)`
  
          Initialize RANLUX with specified luxury
level and seed.

`Ranlux(long ins)`
  
          Initialize RANLUX with default luxury
level and a specified seed.


| **Method Summary** | |
| --- | --- |
| `java.lang.Object` | `clone()`             Returns a copy of the receiver; the copy will produce identical sequences. |
| `double` | `raw()`             Returns a uniformly distributed pseudo-random double in the range (0,1). |
| `void` | `setDiag(boolean b)`             Turns diagnostic messages on and off. |

**Methods inherited from class edu.cornell.lassp.houle.RngPack.RandomSeedable**

`ClockSeed, ClockSeed`

**Methods inherited from class edu.cornell.lassp.houle.RngPack.RandomElement**

`choose, choose, gaussian, gaussian, powlaw, raw, raw, uniform`

**Methods inherited from class java.lang.Object**

`equals, getClass, hashCode, notify, notifyAll, toString, wait, wait, wait`

**Field Detail**

### lxdflt

```
public static final int lxdflt
```

:   Default luxury level: `lxdflt=3`

    **See Also:**: Constant Field Values

---


### maxlev

```
public static final int maxlev
```

:   Maximum luxury level: `maxlev=4`

    **See Also:**: Constant Field Values


**Constructor Detail**

### Ranlux

```
public Ranlux()
```

:   Default initialization of RANLUX. Uses default seed
    `jsdflt=314159265` and luxury level 3.

---


### Ranlux

```
public Ranlux(java.util.Date d)
```

:   Initialize RANLUX with default luxury
    level and a Date object. Can be used to conveniently initialize
    RANLUX from the clock,

    ```
     RandomElement e = Ranlux(new Date());
    ```

    **Parameters:**: `d` - date used to generate seed

---


### Ranlux

```
public Ranlux(int ins)
```

:   Initialize RANLUX with default luxury
    level and a specified seed.

    **Parameters:**: `ins` - seed, a positive integer

---


### Ranlux

```
public Ranlux(int lux,
              java.util.Date d)
```

:   Initialize RANLUX with specified luxury
    level and a Date object. Can be used to conveniently initialize
    RANLUX from the clock,

    ```
     RandomElement e = Ranlux(4, new Date());
    ```

    **Parameters:**: `lux` - luxury level from 0-4.: `d` - date used to generate seed

---


### Ranlux

```
public Ranlux(int lux,
              int ins)
```

:   Initialize RANLUX with specified luxury
    level and seed.

    **Parameters:**: `lux` - luxury level from 0-4.: `ins` - seed, a positive integer.

---


### Ranlux

```
public Ranlux(int lux,
              long ins)
```

:   Initialize RANLUX with specified luxury
    level and seed.

    **Parameters:**: `lux` - luxury level from 0-4.: `ins` - seed, a positive long.

---


### Ranlux

```
public Ranlux(long ins)
```

:   Initialize RANLUX with default luxury
    level and a specified seed.

    **Parameters:**: `ins` - seed, a positive integer


**Method Detail**

### clone

```
public java.lang.Object clone()
```

:   Returns a copy of the receiver; the copy will produce identical
    sequences. After this call has returned, the copy and the receiver have
    equal but separate state.

    :   **Overrides:**: `clone` in class `PersistentObject`
    :   **Returns:**: a copy of the receiver.

---


### raw

```
public final double raw()
```

:   Returns a uniformly distributed pseudo-random double in the range (0,1).

    :   **Specified by:**: `raw` in class `RandomElement`
    :   **Returns:**: a uniformly distributed pseudo-random double in the range (0,1). **See Also:**: `RandomJava`

---


### setDiag

```
public void setDiag(boolean b)
```

:   Turns diagnostic messages on and off. If setDiag(true) is
    called, RANLUX will print diagnostic information to
    System.err

    :   **Parameters:**: `b` - diagnostic message status


---


|  |  |  |
| --- | --- | --- |
| **Overview** \| **Package** \| **Class** \| **Use** \| **Tree** \| **Deprecated** \| **Index** \| **Help** | |  |
| **PREV CLASS**   **NEXT CLASS** | **FRAMES**    **NO FRAMES**     **All Classes** |  |
| SUMMARY: NESTED \| FIELD \| CONSTR \| METHOD | DETAIL: FIELD \| CONSTR \| METHOD |  |


---
